# Table of Diarrhœa Symptoms, with Remedies

**Published:** 1882-03

**Authors:** C. Hering


					﻿TABLE OF DIARRHOEA SYMPTOMS, WITH REMEDIES.
We reprint a table of diarrhoea symptoms, with remedies, prepared
and published many years ago, by C. Hering. We have a number of
them on various subjects, dyspepsia, headache, toothache, etc., which
we will publish later. In this table the characteristic symptoms of a
few remedies are presented; for hasty reference nothing can be better.
I • • 11	I I -I 1. -M ’I 1 II I I I Is'
«	. to •£ i i	I ci I . I • I i i r£ ii 1—s o i E fa I	w •	. Q . <4 P
d 3	• o	I I fl : 9 c I d o i. «< ' • iS IC *"i * 5 P . 2 _fl • I #4 0 ctf co .	*4 E
i ib i ’i i 1 iLb i §LW-3 g § s g o f < if sH-g o ibib- * J o s f HgNtHikWl §
o bo c £ £ 4> >_ a w « j	o o o o a j- 3 <u £ SJ R *8 « >» £ LS e ® ft lfl la 2 s W W S’ ex, 2 ins i a 3 2 «
1) EVACUATIONS	**"	® ® O O Q OjU |O. O IO 1O |O O Q Q O 5C |>h •■* |M j j ,2 Z Z Z O Ok Cu SU Cu Pi 04 co co co co co co co
» streaked with blood.....................■.L..L..I...'.... _ ... ...I..'...!»..L.J... ..J...... ..................I......	...i—|i^...	_ ... _ ... ......I...... ...I.......
bloody and slimy........................................................................................................................................   .........	—.........
2 like pap...................................j.............................. .............. ... —........................................................           _	_ — ......
2 | like corruption.......................j...|... ...LJ...|... ... ... ... ... '‘w^L*' y*’r”	•••!••• ••• ••• ... ... ...
g smelling offensive.........................I...:... ... _	...|...i... ... ... ... ... ... ... ... ... ...i... ... ... ... ...|_i..-1... —I —i____... ... ...i... _I —-j... ...
Q undigested...............................    ...^_____... _ ...L.. _ ..A_J...1...... ... ... ... ... ...L_L^L........ ...I... ...|—ka	.................. ... ...
“ with vomiting..............................—	...... ... ... ...	... ... ... ... ... ... ... _...... ... ... ..*.	' — i...I... ... ...I...	_
“ after overheating...................................................................................   _	............4 ... _ .............................................
“ after taking cold........................I.......— ...j...	I...I.......1X1.____________
“ without pain.......................I......LaLo'...	— I... I...|— ..'3u. ..J...... ... ... ... ••• — I—. ... ... ... ... — .Li.... — ...I... ...I...L.. ... ... ..J...	.
“ weakening.............................. . .. .. .. .. _	.j.._i...	4'...	...I...I... ... ... ... ""j...
Inclination to stool without evacuation... _	____... ... ... ...L_ — •••’ — • ••••• .................. ...h... — | — M ... — ...L.l,,.	...L. ...______... ... -. —.Li
2) ACCOMPANYING SYMPTOMS.
a)	BEFORE HAVING A PASSAGE:
Chilliness......................................... .. .. .. . .. .. .. .. .. .. . .. .. .. .. ...|... ...k.. ... ... ... ... ...... — .1 ... ... ... ... ...... ... ......	... ... ... _
Nausea....................................... ............................1.—
b)	WHILE HAVING A PASSAGE:
Burning in the anus.......................1...1... ... ... _ ...-------...; —I... ...	—_j... ... ... ...I-------------------4	—
Pain in the anus..........................I...I... — ...1;.. ...I.,.|...	... ...|... ... ... ... ...I... ...i...|... _i_ ...|...l... ...I —	...1... ... ... ...I...	...... ... ... ... ...
Discharge of wind.........................
Thirst....................................... ......L..	—i — I... .................. ...I... I...,......... ...... ...... .y|... ..................... ...... .................. ...
Nausea.....................•..............I..J... _ ... ...I...I../... ... ... ... ... ... ... ... _;... ... ... ... ... ... — ...t... ...J — .4 — 1... ... ... ... ... ... ... ... ... ... ... ......L_ ... —
Vomiting.................................... I... <......... _	...... ......j......... ...... I — ......... •••kfr — ••••••
Perspiration..............................|...k..:...k..;...|^,...|...	..-.i...	...1... ...'— .t.,...|...	...1...
c)	AFTER HAVING HAD A PASSAGE : j
Burning in the anus...........................  .i.	_	— ••• ...j...	...1... ..J...	_________;...i—
Vomiting..................................j...L..>..4. ...i... ...|...b..>"|.>.H..i...|... ... ... ...L.. ...l...i...l... ... ... ...I... ...i••• .•• .f.:...|...i... ... ...I...I...I...I...	...i —
Pain in the abdomen............;..........^rp..|...p.............. —k*.	~	...L_	L.L..|— •••	--------—
Inclination to stool....................    ...L.'i	... ... _	'•••: — 1...1...	|... ... ... ......j — ;... ... ... ... ...’— ...
Nausea.................................... ............................ .....................................................’ .....................................|... |.  ......_
_ Marks Symptoms.
				

